# The J-IDEA Pandemic Planner: A Framework for Implementing Hospital Provision Interventions During the COVID-19 Pandemic

**DOI:** 10.1097/MLR.0000000000001502

**Published:** 2021-01-21

**Authors:** Paula Christen, Josh C. D’Aeth, Alessandra Løchen, Ruth McCabe, Dheeya Rizmie, Nora Schmit, Shevanthi Nayagam, Marisa Miraldo, Paul Aylin, Alex Bottle, Pablo N. Perez-Guzman, Christl A. Donnelly, Azra C. Ghani, Neil M. Ferguson, Peter J. White, Katharina Hauck

**Affiliations:** * MRC Centre for Global Infectious Disease Analysis and Abdul Latif Jameel Institute for Disease and Emergency Analytics; † Department of Economics & Public Policy, Centre for Health Economics & Policy Innovation, Imperial College Business School; ‡ Dr Foster Unit, Department of Primary Care and Public Health; § NIHR Health Protection Research Unit in Healthcare Associated Infections and Antimicrobial Resistance, Imperial College London, London; ∥ Department of Statistics, University of Oxford, Oxford; ¶ NIHR Health Protection Research Unit in Modelling and Health Economics, Imperial College School of Public Health; # Modelling and Economics Unit, National Infection Service, Public Health England, London, UK

**Keywords:** COVID-19, hospital provision interventions, adult critical care, health systems capacity, pandemic response, hospital capacity, critical care

## Abstract

**Background:**

Planning for extreme surges in demand for hospital care of patients requiring urgent life-saving treatment for coronavirus disease 2019 (COVID-19), while retaining capacity for other emergency conditions, is one of the most challenging tasks faced by health care providers and policymakers during the pandemic. Health systems must be well-prepared to cope with large and sudden changes in demand by implementing interventions to ensure adequate access to care. We developed the first planning tool for the COVID-19 pandemic to account for how hospital provision interventions (such as cancelling elective surgery, setting up field hospitals, or hiring retired staff) will affect the capacity of hospitals to provide life-saving care.

**Methods:**

We conducted a review of interventions implemented or considered in 12 European countries in March to April 2020, an evaluation of their impact on capacity, and a review of key parameters in the care of COVID-19 patients. This information was used to develop a planner capable of estimating the impact of specific interventions on doctors, nurses, beds, and respiratory support equipment. We applied this to a scenario-based case study of 1 intervention, the set-up of field hospitals in England, under varying levels of COVID-19 patients.

**Results:**

The Abdul Latif Jameel Institute for Disease and Emergency Analytics pandemic planner is a hospital planning tool that allows hospital administrators, policymakers, and other decision-makers to calculate the amount of capacity in terms of beds, staff, and crucial medical equipment obtained by implementing the interventions. Flexible assumptions on baseline capacity, the number of hospitalizations, staff-to-beds ratios, and staff absences due to COVID-19 make the planner adaptable to multiple settings. The results of the case study show that while field hospitals alleviate the burden on the number of beds available, this intervention is futile unless the deficit of critical care nurses is addressed first.

**Discussion:**

The tool supports decision-makers in delivering a fast and effective response to the pandemic. The unique contribution of the planner is that it allows users to compare the impact of interventions that change some or all inputs.

## Abstract 2

Supplemental Digital Content is available in the text.

The coronavirus disease 2019 (COVID-19) pandemic challenges existing hospital care capacity due to a surge in demand, particularly for critical care (CC) and respiratory support. Peak demand may exceed existing supply in many countries. Preparing hospitals first requires a rapid assessment of the existing capacity of the health system, to determine the baseline from which subsequent hospital provision interventions increase capacity.^1^ Decision-makers must rapidly decide on interventions that optimize hospital capacity to address the demand for care. Additional capacity to accommodate COVID-19 patients may be obtained by demand-side or supply-side interventions; an example of the former is cancelling elective surgery, whereas examples of the latter are rapidly constructed field hospitals, and recruitment of retired health care staff.

A variety of tools have been developed to estimate bed occupancy^2^ and calculate capacity requirements with respect to forecasts of COVID-19 patients.^3^–^5^ Each tool is relevant at different stages of a country’s epidemic and helps to answer different questions. The COVID-19 Hospital Impact Model for Epidemics assists hospital capacity planning during the period before a region’s peak infections by estimating the number of hospital beds required.^3^ The CDC COVID-19 Surge tool^4^ and the World Health Organization Essential Supplies Forecasting Tool^5^ can be used to estimate the surge in demand for hospital-based services and compare this with existing or expanded resources. However, having estimated demand it is necessary to determine how to provide the capacity to meet that demand, and none of these tools provide quantitative estimates of the increases in capacity provided by alternative intervention options. A rapid-use estimation tool is required to weigh different intervention options to plan surge capacity for epidemic peaks, and plan scaling-down afterwards. This is the purpose of the Abdul Latif Jameel Institute for Disease and Emergency Analytics (J-IDEA, Supplemental Digital Content 1, http://links.lww.com/MLR/C182) pandemic planner, as described in this paper. It is a practical and user-friendly tool targeted at hospital administrators, commissioners, national policymakers, and other decision-makers, while being based on solid research evidence on the impact of interventions.

Here, we present this publicly available planning tool, providing information on how key variables are defined, exemplifying how it can be used in a case study of English field hospitals, and discussing the key limitations and challenges to consider.

## METHODS

### Tool Development

To inform the design of the tool, we identified hospital capacity interventions that were implemented or considered by 12 European countries in March and April 2020 as the demand for COVID-19 health care increased. These interventions are implemented at hospital or national level, and manage admissions of patients to care, reorganize and increase capacity, and more subtly adapt care processes and patient pathways. The information was collated from national health ministry Web sites, health agencies, news media and the European Observatory’s Health System Response Monitor, and consolidated in an interactive map (https://microreact.org/project/9iAtQhHL6). We identified 18 interventions, of which 13 increase or reorganize the provision of care and 5 manage admissions to care, particularly CC. The 11 most common interventions were included in the planner (Table 2, Appendix A, Supplemental Digital Content 2, http://links.lww.com/MLR/C183); of those increasing or reorganizing the provision of care, this included providing additional beds in rapidly constructed field hospitals (10 countries), repurposing general and acute (G&A) beds into CC beds (7 countries), converting operating theaters into CC wards (7 countries), deploying newly qualified and final year medical and nursing students (9 countries), deploying former health care staff (11 countries), deploying international doctors at the final stage of their conversion assessment (ie, expedite accreditation of doctors who have qualifications from overseas) (4 countries), use of private hospital resources (6 countries), upskilling staff (5 countries), and procurement or donations of additional medical equipment (11 countries). Additional interventions may become necessary during extreme pandemic surges that prioritize scarce resources among patients requiring urgent care: we found that all countries planned on cancelling planned nonurgent, noncancer elective surgery, and most considered national guidelines addressing admissions thresholds to CC or had these in place already (10 countries). These 11 included interventions have trade-offs with respect to additional staff requirements, reductions in quality of care, financial challenges, and others which are outlined in Table 2.

Nearly all countries also increased provision of personal protective equipment, but this was outside the scope of this planner which is more broadly concerned with hospital interventions that increase or reorganize care and manage demand. Calculation of personal protective equipment requirements is addressed by other tools.^5^,^6^

### Modeling Strategy

From the information collated, we identified 3 key resources that were being reorganized or increased: staff, beds, and respiratory support equipment. In addition, we made the distinction between CC and G&A wards, as they vary in the required skill set of staff, essential equipment (respiratory support), and staff to beds ratios. For the purpose of this study, we made no distinction between the terms “Critical Care” (CC) wards and “Intensive Care Units” used in some countries (eg, the United States).

Nurses and medical doctors were stratified by ward in which they primarily work. The medical doctor category is split further into senior and junior staff, reflecting the requirement of a senior clinical decision-maker responsible for the care of a group of patients (ward-based). For example, a junior doctor is similar to a medical resident or fellow, and a senior doctor similar to an attending physician in the United States. Staff categories were measured in full-time work equivalents, which accounts for any staff employed on a part-time basis or absent due to non-COVID-19 illness. In some countries, absence from work due to COVID-19 is high among medical staff, therefore the tool incorporates COVID-19 absences separately from normal absences.

To understand the impact of the hospital interventions, baseline inputs of existing (or prepandemic) hospital resources are required. The planner leaves it to the user to set baseline capacity: it could reflect prepandemic baseline capacity or consider a baseline that has already been enhanced by multiple interventions. From this baseline, the planner helps users to understand how additional interventions will affect current capacity in care provision. For each intervention, the planner takes as input the difference in capacity from the baseline input of each resource category, expressed as an absolute number and as a percentage difference. Specifically, each intervention increases a resource category (eg, beds) and the planner then calculates the required increase in the other resource categories (eg, staff) that arise from resource dependencies (eg, staff required to operate new beds applying a specific staff-to-bed ratio). Not considering such dependencies can reduce the effectiveness of interventions. As such, the tool allows users to take a comprehensive view of capacity constraints considering all crucial resources and make informed choices for enhancing capacity.

The uncertainty in baseline capacity, COVID-19 patients and intervention effect are not explicitly modeled in the tool but can be explored using a scenario-based analysis. The planner is designed to facilitate such scenario-based analyses, the specifics of which are left to discretion of the user. By varying different inputs, such as reasonable best- and worst-case numbers of COVID-19 patients in G&A and CC, the user can understand any capacity constraints under different scenarios and the optimal interventions to mitigate these.

## RESULTS

The J-IDEA pandemic planner can be found in additional file 1, accompanied by a detailed user guide in Appendix B, Supplemental Digital Content 2 (http://links.lww.com/MLR/C183). In addition, a video of the tool is publicly available,^7^ and the authors hosted an online webinar to demonstrate the tool and answer questions from the general audience.^8^

### Calculation of Capacity

The tool allows hospital administrators, commissioners, national policymakers, and other decision-makers to quantify the impact on hospital capacity of different intervention options to enable planned upscaling (and downscaling) capacity to meet changing demand for care. The unique contribution of the planner is that it allows users to compare the impact of interventions that change some or all inputs. It calculates capacity under different intervention scenarios, identifying the limiting factors in hospital capacity for each scenario. Further, the tool informs which interventions provide the greatest improvement across inputs, whether a combination of interventions can be leveraged to change capacity, and under what circumstances interventions can be scaled down. Given the user-specified number of patients, the planner calculates the spare capacity in terms of beds, staff, and respiratory support equipment, either in absolute terms or per 10,000 population for each health care capacity intervention, with a negative number representing a deficit in capacity. This output is also reported graphically per input and per intervention. In addition, the percent change in spare capacity compared with the baseline is calculated to allow for the comparison of different interventions, and staff-to-bed ratios, and to quantify the maximum number of beds per category of staff required to provide care safely (further detailed in Appendix B, Supplemental Digital Content 2, http://links.lww.com/MLR/C183). Under these ratios, the tool can also be used to identify whether the current staff capacity is sufficient to treat all patients (both COVID-19 and non-COVID-19) once bed capacity is reached, or whether additional staff are required to safely implement the intervention.

### User Inputs

Assessment of the existing hospital care capacity establishes the baseline before any efforts to increase capacity are implemented. The tool focuses on hospital care inputs that are required for the treatment of COVID-19 and similar conditions, that is acute respiratory distress. Data on the current staff (numbers and absences), whether staff are trained in CC, bed numbers, and respiratory support equipment (eg, ventilators or continuous positive airway pressure devices) must be collected. All data must consider the appropriate scale, which could be at a national, local or single hospital level: the planner is adjustable to the health care system of interest.

The user is required to input baseline and COVID-19-related variables (Table 1). The baseline capacity and patient occupancy can be determined using data from a defined period during nonpandemic times which is representative of prepandemic hospital care capacity; we give further recommendations on how to estimate baseline capacity patient demand in the user guide Appendix B Section 3 (Inputs), Supplemental Digital Content 2, http://links.lww.com/MLR/C183. Users can input the number of expected COVID-19 patients to estimate how their baseline spare capacity, which is a function of the baseline capacity and patient occupancy, will be impacted by these additional patients. By varying the inputs of expected COVID-19 patient numbers, the user can explore the impact of an upwards (peak to come) or downwards (peak has passed) trajectory and thus whether scaling up or down is appropriate. Further, users can change the assumptions of the impact of the incorporated intervention options based on their country’s care provision and circumstances, as well as extend the tool to model additional interventions.

**TABLE 1:** Summary of Resource Inputs Required from the User

Variable	Baseline	COVID-19	Hospital Interventions (Changes in Values)
Beds			
CC beds available	X		X
CC beds occupied by non-COVID-19 patients	X		X
CC beds occupied by COVID-19 patients		X	
G&A beds available	X		X
G&A beds occupied by non-COVID-19 patients	X		X
G&A beds occupied by COVID-19 patients		X	
Operating theaters available			X
Beds per operating theater			X
Staff			
CC nurses	X		X
CC senior doctors	X		X
CC junior doctors	X		X
CC nurse per bed	X		
CC senior doctor per bed	X		
CC junior doctor per bed	X		
G&A nurses	X		X
G&A senior doctors	X		X
G&A junior doctors	X		X
G&A nurse per bed	X		
G&A senior doctor per bed	X		
G&A junior doctor per bed	X		
Nurse sickness rate		X	
Doctor sickness rate		X	
Equipment			
No. breathing equipment available	X		X
Non-COVID-19 patients requiring equipment	X		X
COVID-19 patients requiring equipment		X	
Other			
Staff FTE multiplier	X		
Reference population size	X		

CC indicates critical care; COVID-19, coronavirus disease 2019; FTE, full-time equivalent; G&A, general and acute; X, required input.

The planner is prepopulated with the baseline capacity and estimated intervention impacts for England; the methods used are detailed in Appendix B1 and B2, Supplemental Digital Content 2 (http://links.lww.com/MLR/C183).

### Case Study: Implementation of field Hospitals in England

We demonstrate the tool with a case study of England, which had 154,258 confirmed COVID-19 cases by June 5, 2020^9^ and consequently had to quickly review, redistribute, and expand health care capacity.

The prepandemic baseline of capacity in England was determined using publicly available data on hospital resources.^10^,^11^ At baseline there were 99,569 G&A beds, of which 90% were occupied, whereas there were 4114 CC beds of which 80% were occupied (Appendix Table B1, Supplemental Digital Content 2, http://links.lww.com/MLR/C183). For CC staff, there were an estimated 3939 nurses, 965 senior doctors, and 677 junior doctors in full-time work equivalents, whereas for G&A wards there were 32,354 nurses, 12,680 senior doctors, and 10,293 junior doctors (Appendix Table B1, Supplemental Digital Content 2, http://links.lww.com/MLR/C183).

**FIGURE 1:**
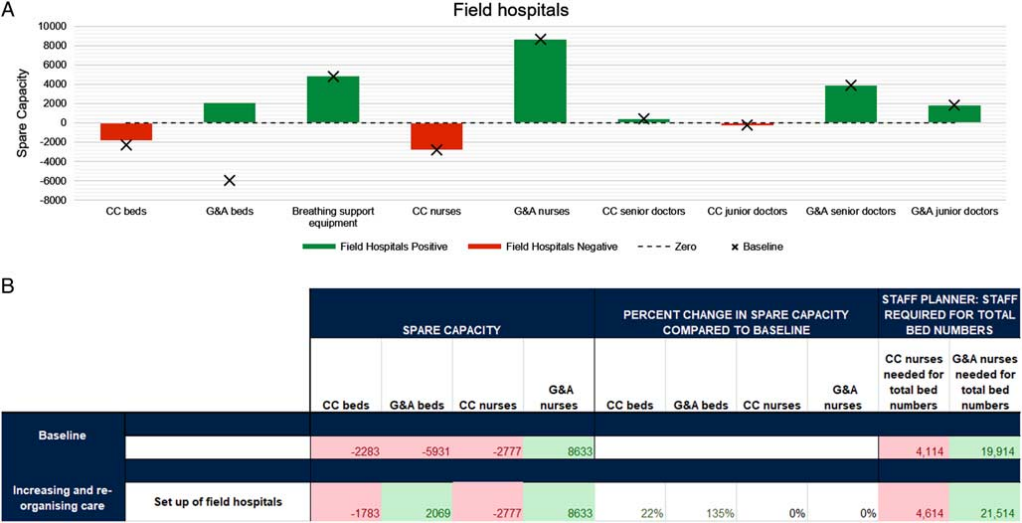
Impact of field hospitals with 3100 critical care (CC) and 15,700 general and acute (G&A) COVID-19 patients. A, Comparison of spare capacity of modeled resources with implementation of field hospitals. B, Numeric output provided by the planning tool, showing spare capacity of CC and G&A beds and nurses under the baseline and with field hospitals, as well as the percent change in spare capacity compared with baseline and the number of nurses needed to staff the provided bed numbers (columns for other resources have been omitted).

National Health Service (NHS) “Nightingale” field hospitals were set up in England in March 2020. It is estimated that this increased the number of CC and G&A beds by 500 (12%) and 8000 (8%), respectively.^12^,^13^ As of June 5, 2020, the observed maximum number of hospitalized COVID-19 patients on any day in England were 3100 in CC and 15,700 in G&A care. Using the planning tool, we estimated that with field hospitals England would have 2069 spare G&A beds, compared with a deficit of 5931 at the baseline. This estimate considers hospitalized COVID-19 patients on top of the average baseline occupancy of 89,800 non-COVID-19 patients in G&A. This intervention would allow enough staff to safely cover all G&A beds in accordance with guidelines (Fig. 1).^14^

However, even with field hospitals, a deficit of 1783 CC beds would remain when considering existing occupancy, although the magnitude of the deficit is reduced by 22% from the baseline. According to the specified staffing ratios,^15^ an additional 500 CC nurses would be required to safely operationalize the new CC beds in the field hospitals (Fig. 1). This result highlights that other interventions to increase CC beds are futile unless the deficit of CC nursing is addressed first. The planner shows where limitations in hospital capacity exist, which is crucial for reorganizing care for any future pandemic surges.

**FIGURE 2:**
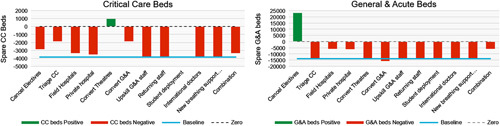
Comparison of the impact of different interventions on spare capacity of critical care (CC) and general and acute (G&A) beds for 4650 CC and 23,550 G&A COVID-19 patients in the English hospitals case study.

We examined additional scenarios which hospital planners may wish to consider for the second wave of the pandemic. For example, 1 scenario could be that the peak demand in a second wave will be half that observed in the first wave (ie, 7850 and 1550 COVID-19 patients in G&A and CC, respectively). Under this assumption, the deployment of field hospitals continues to allow for the safe treatment of all G&A patients, but the deficits in CC resources under the specified staffing ratios persist. Although this is a reduction on that which was estimated previously (estimated deficit of 233 CC beds and 1227 CC nurses), it underlines a more widespread issue with surge capacity of CC resources in England. An alternative scenario could be that the peak demand in a second wave will be 50% higher than that observed in the first wave (ie, 23,550 and 4650 COVID-19 patients in G&A and CC, respectively). In this scenario, despite the extra capacity of beds from NHS Nightingale, there are expected deficits in both CC and G&A resources. This indicates that this intervention alone is not sufficient to provide hospital care to this hypothetical number of COVID-19 and non-COVID-19 patients. The plots produced by the tool show the impact of all modeled interventions on spare capacity of the different resources, highlighting that only conversion of operating theaters and cancellation of electives on their own could address the respective deficits in CC and G&A beds under this scenario (Fig. 2).

## DISCUSSION

We have developed the J-IDEA pandemic planner to assist health care planning for extreme surges in demand for hospital care for COVID-19, while retaining capacity for other conditions. The tool is suitable for use at a variety of levels from national policymaking to planning at regional level or for individual hospitals. It is designed to estimate demand for COVID-19 care by enabling users to examine different intervention options to increase hospital capacity, if required, to meet a surge in demand and then to scale-down after the epidemic peak.

The tool is widely applicable: although our choice of hospital provision interventions was informed by a rapid review of European countries, their implementation has not been limited to high-income settings. Various middle-income countries have utilized similar measures. For example, South Africa, Turkey, and Kenya introduced field hospitals.^16^–^18^ Similarly, retired medical professional and medical students were deployed in India and Peru.^19^,^20^

In the case study for England the respiratory support equipment is ventilators, whereas in other settings, the relevant respiratory support equipment may be continuous positive airwave pressure devices, and the staffing ratios may be different. A particular challenge for user inputs is a potential lack of publicly available national data to accurately quantify the increase to capacity arising from the implementation of hospital provision interventions. Nonetheless, our case study highlights how even imperfect data can be used as a proxy to assess the validity of the framework. Furthermore, where users are managers of individual hospitals, they will have access to the relevant information locally. Although the tool provides a simple and flexible way to compare the impact of different hospital provision interventions on capacity, we acknowledge the inevitable limitations of emergency interventions for health care provision,^21^ some of which are explored in Table 2. The reviewed interventions are associated with either difficult ethical decisions surrounding the prioritization of care for certain patient groups,^22^ large financial costs to sustain these efforts^23^ or trade-offs between quantity and quality of care provided.^24^ Of course, planners will need to consider the opportunity costs of the interventions and against each other, or indeed against keeping capacity at baseline. Patient admission type, non-COVID-19 patient numbers, and variation in length-of-stay may have nontrivial effects on capacity, but the data to evaluate these effects are currently unavailable. As such, our tool does not estimate the number of patients that can be accommodated for different conditions at any time but rather the number of patients that can be accommodated at any time. However, the application of the tool explores alternative ways of increasing capacity and allocating scarce resources during the COVID-19 pandemic and contributes to a wider set of tools needed by decision-makers during a pandemic.

**TABLE 2:**
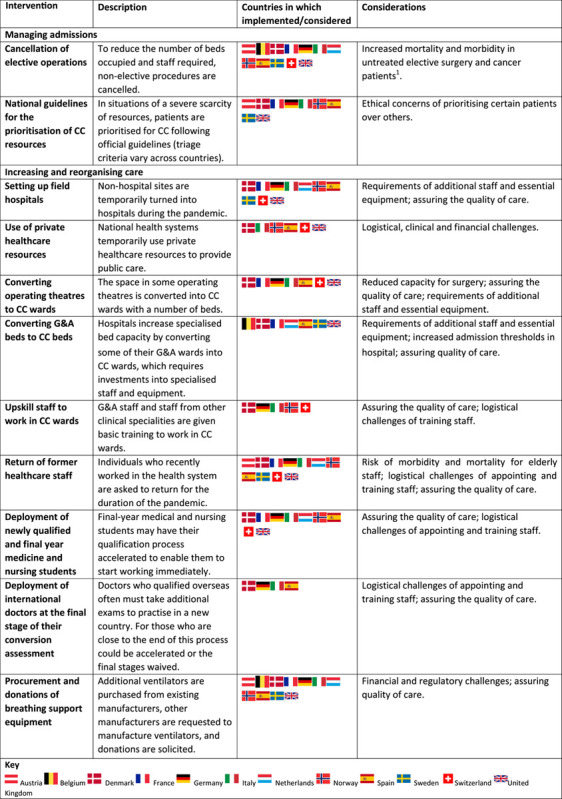
Overview of Hospital Interventions to Manage Admissions and to Increase and Reorganize Care

^1^ https://bmcpublichealth.biomedcentral.com/articles/10.1186/s12889-019-6526-6; https://pubmed.ncbi.nlm.nih.gov/27299977/.

Demand for health care needs to be reassessed continuously, including from non-COVID-19 patients. Admissions of patients with other conditions may change during the pandemic. Patients may not seek hospital care, either because they fear hospital-acquired COVID-19 infection or because they do not want to overburden hospitals. The patterns of presenting conditions may change due to widespread public health (ie, epidemic) interventions, for example, lockdown policies may reduce road traffic accidents.^25^,^26^

In addition to using the planner, decision-makers also need to consider the potential wider health impact of interventions. For instance, increasing the number of beds without corresponding increases in staffing will reduce staff-to-beds ratios may adversely impact the quality of care.^27^ Cancelling elective operations will increase morbidity, and potentially mortality, in the affected patients.^28^,^29^

Admissions thresholds may imply that not all patients who could potentially benefit from life-supporting CC are able to receive it.^30^,^31^ Prioritizing patients for admission to hospital care is associated with complex bioethical considerations. Policies need to be assessed carefully with respect to existing guidelines, such as those published by the World Health Organization,^32^,^33^ the African Federation for Emergency Medicine,^34^ the UK National Institute for Health and Care Excellence in preparation for the COVID-19 pandemic,^21^ or those of the Società Italiana di Anestesia Analgesia Rianimazione e Terapia Intensiva in Italy.^35^ The guidelines are based on well-established literature.^22^,^36^,^37^

The planner helps inform choices in the preparation of hospitals for the pandemic. The current format has been chosen to make it as widely usable as possible, requiring minimal inputs, assumptions, and technical expertise from the user. It also allows the user to tailor parameters to specific health systems. Coordination and capacity planning can improve response efficiency, promote a sense of global security and support, and, ultimately, save lives.

## Supplementary Material

SUPPLEMENTARY MATERIAL Supplemental Digital Content is available for this article. Direct URL citations appear in the printed text and are provided in the HTML and PDF versions of this article on the journal's website, www.lww-medicalcare.com.
