# Supplementary material for: The J-IDEA Pandemic Planner: A Framework for Implementing Hospital Provision Interventions During the COVID-19 Pandemic
Source: Med Care. 2021 Jan 21;59(5):371–8. doi: 10.1097/MLR.0000000000001502 (PMC7610624; doi:10.1097/MLR.0000000000001502)
Supplement: SUPPLEMENTARY MATERIAL [file mlr-59-371-s002.docx]

**The J-IDEA pandemic planner: a framework for implementing hospital provision interventions during the COVID-19 pandemic**

**Appendix**

# Appendix A: Country-level intervention sources

|  | **Inclusion in planner** | **Countries that implemented or considering implementation^[[1]](#footnote-1)^** | **References** |
| --- | --- | --- | --- |
|  | | | |
| Cancellation of elective operations | Yes | 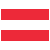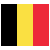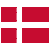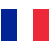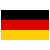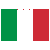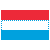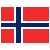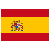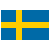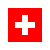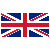 | ^1–12^ |
| National guidelines for the prioritisation of CC resources | Yes | 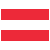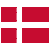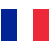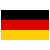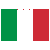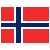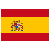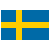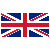 | ^4,9,11,13–18^ |
| Relocation of patients to hospitals in bordering countries | No | 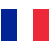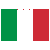 | ^19^ |
| Continuity of care homes for the treatment of the elderly | No | 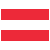 | ^20^ |
| Set up of patient management and triage strategies | No | 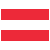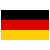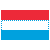 | ^21–24^ |
|  | | | |
| Setting up field hospitals | Yes | 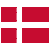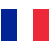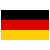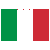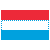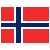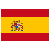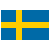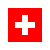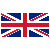 | ^22,25–33^ |
| Use of private healthcare resources | Yes | 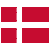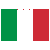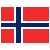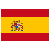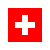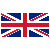 | ^1,34–38^ |
| Converting operating theatres to CC wards | Yes | 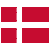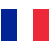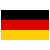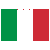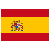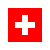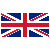 | ^1,39–44^ |
| Converting G&A beds to CC beds | Yes | 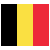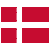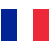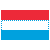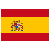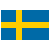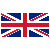 | ^8,22,34,39,40,45–50^ |
| Upskill staff to work in CC wards | Yes | 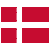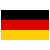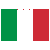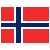 | ^22,34,51,52^ |
| Return of former healthcare staff | Yes | 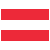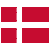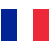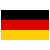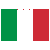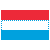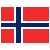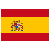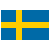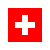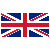 | ^22,39,53–61^ |
| Deployment of newly qualified and final year medicine and nursing students | Yes | 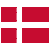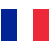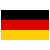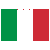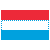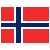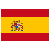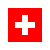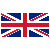 | ^22,34,49,56–60,62,63^ |
| Deployment of international doctors at the final stage of their conversion assessment | Yes | 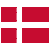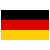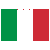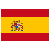 | ^22,39,49,64^ |
| Procurement and donations of respiratory support equipment | Yes | 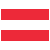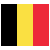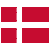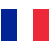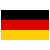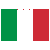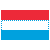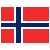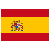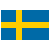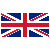 | ^4,65–74^ |
| Procurement and donations of additional Personal Protective Equipment (PPE) | No | 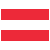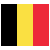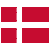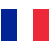 | ^22,34,47,68,75–80^ |
| Efficient redistribution of PPE across hospitals | No |  | ^34,79,81–87^ |
| Export ban on PPE | No |  | ^88–92^ |
| Financial aid to hospitals to purchase additional resources | No |  | ^93–96^ |

# Appendix B: J-IDEA pandemic hospital planner user guide

1. Introduction

The tool allows users to enter their forecasts on the number of expected of COVID-19 and other patients requiring care, to compare potential deficits in capacity on a particular day. The tool presents a snapshot of healthcare capacity. Users are advised to read *Section 5 (Modelling Assumptions)* and whether the modelled interventions and assumptions underlying the calculations meet their setting-specific requirements. Assumptions which do not apply to the setting of interest can be changed by the user. The following guide can be used to understand each component of the tool, underlying assumptions and how results can be retrieved.

The tool can be found on the COVID-19 repository belonging to Imperial College London’s MRC Centre for Global Infectious Disease Analysis. (URL: <https://www.imperial.ac.uk/media/imperial-college/medicine/mrc-gida/2020-04-17-COVID19-Report-15-hospital-planner.xlsm>). A seminar with a Q&A was conducted and can be found here: [https://www.youtube.com/watch?v=Y-rlUoylR9c](https://www.youtube.com/watch?v=Y-rlUoylR9c&feature=youtu.be).

1. How to use this tool

The planner is implemented in a spreadsheet consisting of 5 sheets:

- Readme: provides an overview of the tool and the different hospital capacity interventions to model.
- Input (Quick): the most inputs to be entered by the user.
- Input (Extended): an extended version of *Inputs (Quick)*, that includes data specific to the health care system, to be entered by the user, if assumptions for the setting of analysis differ.
- Output (numeric): numeric results and breakdown of calculations.
- Output (graphical): graphically illustrated results.

The inputs, outputs and modelling assumptions are detailed in the following sections, accompanied by a case study focusing on England for illustration. The planner is pre-populated with values for England. The tool is designed to be flexible and applicable at different healthcare levels and in various settings, as the interventions have been implemented in middle-income settings as well ^97–101^. Users must decide whether the opportunity costs or ethical implications of the hospital interventions align with societal norms. For more information on altering intervention assumptions, refer to *Section 3b (Editable intervention assumptions)*.

A Quick User Guide with step-by-step instructions and an example application is also provided with the planner.

1. Inputs

Users can choose to fill in the *Input (Quick)* or Input *(Extended)*. *Input (Quick)* highlights only the mandatory baseline and intervention values required to use the tool based on the modelling assumptions set out in Appendix Tables B1 and B2. Values entered in *Input (Quick)* are automatically copied across into the appropriate column in *Input (Extended)*. If *Input (Quick)* has blank cells for these values, then the default England value is used instead. *Input (Extended)* offers a more comprehensive data entry by allowing users to change all inputs and assumptions, allowing customisation of the intervention assumptions underpinning the England example and enabling users to create their own combination of interventions. Note that making changes in this sheet will override the inputs from *Input (Quick)*. To reset *Input (Quick)* functionality, users must press the *Reset Formulae* button on the *Input (Extended)* sheet. This defaults the inputs back to England values*.*

1. Baseline user inputs

Inputs are disaggregated into baseline and COVID-19-related inputs. A description of each input and the estimated values for England are presented in Appendix Table B1.

**Baseline inputs on existing hospital capacity and patient occupancy [Inputs (Extended) cells C16-N16]**

Baseline existing hospital capacity reflects non-COVID-19 related provision of beds, respiratory support equipment (ventilators, in our example analysis), and staff before the modelled capacity management interventions are introduced. Baseline capacity inputs can be completed using pre-pandemic or current data. Baseline patient occupancy data reflects the number of non-COVID-19 patients within the healthcare system, split by those occupying G&A or CC beds, before the pandemic. This data should be from the same time period as the capacity data. If using pre-pandemic data then users should choose the most representative data, e.g. from the corresponding month of the previous year, to account for the impact of seasonality on demand. If using current data then the user needs to know the breakdown of patients hospitalised with COVID-19 or for other reasons and the shift in the original baseline due to interventions that have already been implemented.

The tool uses staffing requirements expressed in full-time equivalents (FTEs) to account for part-time working and absence due to non-COVID related sickness and holiday. FTEs may differ from a simple headcount for this reason. If only staff headcounts are known (not FTEs) then see the *Additional inputs* rows in Appendix Table B1.

These baseline values are required inputs in *Input (Quick)* and can be entered in cells E16:E36.

See Appendix Table B1 for further details on baseline inputs.

**Maximum Staff-to-beds Ratio Allowances [Inputs (Extended) cells O16-T16]**

To account for the fact that beds must be staffed by sufficient staff numbers, the user is asked to specify a maximum threshold of the number of beds that an individual member of staff from each of the 6 categories could safely cover. These thresholds are used to determine the level of staffing required by each intervention, both in terms of the number of patients currently hospitalised and in terms of overall bed capacity.

*Example: if 1 G&A nurse can safely staff a maximum of 5 beds (staff-to-beds ratio = 1:5), the maximum staff ratio allowance input for Beds per G&A nurse [cell P16] is 5.*

These values are assumed to be generalisable across settings and thus not required in *Input (Quick)*.

See Appendix Table B1 for further details on staff-to-beds ratios.

**COVID-19 related inputs [Inputs (Extended) cells C20-G20]**

COVID-19 related inputs can be based on that which is currently observed, or which represents an assumption of future pandemic progression, such as a worst-case scenario. They can also be set to 0 should the user wish to explore the impact of the different interventions for pre-pandemic levels of demand and supply.

These are required inputs in *Input (Quick)* and can be entered there in cells H16: H22.

See Appendix Table B1 for further details on COVID-19-related inputs.

**Additional inputs [Inputs (Extended) cells H20-I20]**

The two additional inputs required by this tool are the FTE staff multiplier and the reference population. The planner by default returns the absolute capacity numbers, if users would rather return the per 10,000 population capacity, they can input a reference population size. This can be input in *Input (Quick)* and can be entered there in cell H24.

See Appendix Table B1 for details on additional inputs.

1. Editable intervention assumptions [columns C-AB, rows 27-43]

In addition to adding the baseline figures, the user can alter the figures in the intervention assumptions section of the tool to better-represent their situation. For example, users can include additional increases in the modelled interventions, or extend the tool to model other interventions or scenarios combining interventions not listed here by inserting rows manually.

Parameters should be added in the respective columns, according to whether they are a figure or a percentage. The calculations in the output sheet come from the percentage change section. If users edit the figures, these are automatically converted into percentages. As described in *Section 5 (Modelling assumptions)*, where the distribution of added capacity between CC and G&A is not known, users can consider either attributing all the resource to one category or distributing the resource in line with the baseline proportions. Calculations for the latter method are already automatically implemented in cells C49:J49, and examples of both proposed solutions are highlighted in Appendix Table B2, with their implementation in the corresponding row of the planning tool. Other allocation assumptions are possible but are not modelled at present.

While *Input (Extended)* gives the user flexibility in modelling interventions, *Input (Quick)* provides only the variables required for each modelled intervention according to the assumptions outlined in Appendix Table B2. These can be entered in columns E-T, rows 43:69 of *Input (Quick)*.

**Note that not all interventions are pertinent to all users, and therefore users should only change the cells relevant to them in either *Input (Quick)* or *Input (Extended)*. The values in *Output (Numeric)* and *Output (Graphical)* are automatically updated based on that which is entered into *Input (Extended)* (either directly or through *Input (Quick)*) and hence should do not require alteration.**

1. Outputs

### Numeric

There are four general categories of outputs: spare capacity [columns C-K]; per cent change in spare capacity compared to baseline [columns L-T]; the number of staff required for total bed numbers [columns U-Z]; and a staff ratio check for current patient numbers [columns AJ, AL, AN, AP, AR, AT]. An overview of the definitions of these and guidance on how they should be interpreted is provided in Appendix Table B3.

A positive value for spare capacity means there is sufficient supply of this resource. A negative value indicates a deficit in capacity, i.e. a lack of this resource required to care for the total number of patients. This is further illustrated in Appendix Figure B1.

**Appendix Figure B1: Illustration of the relationship between user inputs, calculations in the planning tool and outputs.**

### Graphical

Space capacity is also presented graphically, both on a per resource and per intervention basis. Bars are coloured according to whether there is spare capacity or capacity deficit, with the baseline and the zero line also highlighted in each graph.

1. Modelling Assumptions

A summary of the assumptions made in the modelled interventions in the tool can be found in Appendix Table B2. The intervention-specific changes in different capacity components have been estimated for England (Appendix Table B2) and can be adapted by the user (*Section 3b (Editable intervention assumptions)*).

For the interventions involving changes to hospital admissions, the modelled percentage increase in capacity is informed by an analysis of English Hospital Episode Statistics (HES) data ^102^. These values are applied to baseline inputs by reducing the bed occupancy of non-COVID-19 patients entered by the user. For the supply interventions, numeric figures found in government reports and other sources are converted into a percentage increase and applied to the input total capacity figures.

The tool distinguishes between CC and G&A beds and staff of different categories because there are substantial differences in equipment and staffing in these two settings. However, this granularity was not available for each figure in the presented capacity interventions, such as for the deployment of newly qualified nursing students or the use of private hospital resources. In this case, we either attributed all the resource to just one category or, where this was deemed unrealistic, distributed the resource into CC (where appropriate) and G&A according to the corresponding proportions arising from the baseline.

The number of COVID-19 patients is input by the user and represents an addition in demand to the baseline occupancy to which different capacity management interventions are applied. User inputs of COVID-19 related staff sickness rates reduce the total staff FTEs available at baseline and in all interventions.

Under each intervention, the planner calculates the level of staffing and breathing support equipment required to care for a given number of patients based on CC and G&A bed occupancy, which feeds into the calculation of spare capacity (see *Section 4 (Outputs)*). Staff FTE requirements are calculated via multiplication of the staff-to-beds ratios. The number of breathing support equipment required depends on the number of patients in CC, with the input allowing different assumptions for the proportion of CC patients requiring breathing support based on their COVID-19 status.

## Further options for adapting interventions

In many settings, a combination of the modelled interventions has already been implemented in planning for COVID-related surges. In this case, users can also use the tool to explore options for scaling down different hospital interventions as the pandemic starts to tail off. In order to adopt this objective, the baseline numbers need to reflect surge-capacity levels. Specifically, the user should implement the following approach:

- On the *Input (Extended)* sheet, set the baseline inputs of existing hospital capacity [cells C16-N16] to reflect current (upscaled) provision, keeping in mind which combination of interventions this represents
- On the *Input (Extended)* sheet, change the percentages or figures for modelled increases in capacity in the different interventions to the opposite sign (e.g. from positive to negative) [columns C-AB, rows 27-43]. This then corresponds to modelling a *decrease* in capacity from ‘baseline’ (upscaled provision).

For example, in a setting where elective operations have been cancelled and this is reflected in baseline inputs, a user could set *Modelled increase in capacity (%)* for the *Cancellation of elective operations* intervention from 26% to -26% and from 13% to -13% for CC bed and G&A bed occupancy, respectively. In this case, the intervention represents a re-introduction of elective operations.

**Appendix Table B1: Input descriptions, input assumptions and example values for England. Cells references are concerning *Input (Extended)* in the planner.**

| Input variable | Description | Input Assumptions (used to derive input values for England) | England example input value |
| --- | --- | --- | --- |
| Baseline inputs on existing hospital capacity [cells C16-N16] | | | |
| Total number of CC beds | The number of CC beds that exist at baseline. This may also be referred to as “open” beds. It is the sum of CC beds that are occupied by patients and CC beds that could be occupied, regardless but are not. | Adult CC beds for 145 reporting acute trusts (includes intensive care and high-dependency units) ^103^. | 4,114 |
| Total number of G&A beds | The number of G&A beds that exist at baseline. This may also be referred to as “open” beds. It is the sum of G&A beds that are occupied by patients and G&A beds that could be occupied, regardless of Staffing need, but are not. | G&A beds for 152 acute and community provider trusts ^104^. | 99,569 |
| Number of CC beds occupied by non-COVID-19 patients | The number of CC beds that are occupied by patients with non-COVID-19-related conditions at baseline. This can also be calculated by multiplying the average baseline CC bed occupancy rate with the total number of CC beds. | The occupancy given for 145 reporting acute trusts ^103^. | 3,297 |
| Number of G&A beds occupied by non-COVID-19 patients | The number of G&A beds that are occupied by patients with non-COVID-19-related conditions at baseline. This can also be calculated by multiplying the average baseline G&A bed occupancy rate with the total number of G&A beds. | G&A beds occupancy for 152 acute and community provider trusts ^104^. | 89,800 |
| Percentage of non-COVID-19 CC patients requiring respiratory support | The percentage of CC patients with non-COVID-19-related conditions requiring respiratory support.  *If not known, users can use the suggested value of 43%.* | Proxy for the percentage of non-COVID-19 CC patients requiring a ventilator based on analyses in ^105^. | 43% |
| Total number of respiratory support equipment | The number of respiratory support equipment that exist at baseline. It is the sum of currently used devices and devices that could be used on the day but are not.  *If not known, this can be left blank, in which case interventions and outputs relating to respiratory support equipment are not available from the tool.* | As reported in the UK media ^106^. | 8,175 |
| Total CC nurses (FTE) | The full-time equivalent of CC nurses at baseline. | In consultation with staff at Imperial College Healthcare Trust (ICHT), Electronic Staff Records (ESR) data from April – June 2019 were filtered to select nurses usually working in CC wards ^45,107^.  According to the number of beds in each trust, a weighted average of daily FTE was calculated for CC nurses. | 3,939 |
| Total G&A nurses (FTE) | The full-time equivalent of G&A nurses at baseline. | In consultation with staff at ICHT, ESR data from April – June 2019 were filtered to select nurses usually working in G&A wards ^45,107^.  According to the number of beds in each trust, a weighted average of daily FTE was calculated for G&A nurses. | 32,354 |
| Total CC senior doctors (FTE) | The full-time equivalent of senior CC doctors at baseline. | In consultation with staff at ICHT, ESR data from April – June 2019 were filtered to select doctors usually working in CC wards, and if they were able to be classed as clinical decision-makers or not ^45,107^.  According to the number of beds in each trust, a weighted average of daily FTE was calculated for senior CC doctors. | 965 |
| Total CC junior doctors (FTE) | The full-time equivalent of junior CC doctors at baseline. | In consultation with staff at ICHT, ESR data from April – June 2019 were filtered to select doctors usually working in CC wards, and if they were able to be classed as clinical decision-makers or not ^45,107^.  According to the number of beds in each trust, a weighted average of daily FTE was calculated for junior CC doctors. | 677 |
| Total G&A senior doctors (FTE) | The full-time equivalent of senior G&A doctors at baseline. | In consultation with staff at ICHT, ESR data from April – June 2019 were filtered to select doctors usually working in G&A wards, and if they were able to be classed as clinical decision-makers or not ^45,107^.  According to the number of beds in each trust, a weighted average of daily FTE was calculated for senior G&A doctors. | 12,680 |
| Total G&A junior doctors (FTE) | The full-time equivalent of junior G&A doctors at baseline. | In consultation with staff at ICHT, ESR data from April – June 2019 were filtered to select doctors usually working in G&A wards, and if they were able to be classed as clinical decision-makers or not ^45,107^.  According to the number of beds in each trust, a weighted average of daily FTE was calculated for junior G&A doctors. | 10,293 |
| Maximum Staff-to-beds Ratio Allowances Inputs [cells O16-T16] | | | |
| Beds per CC Nurse | The maximum threshold of the number of CC beds that a single CC nurse could safely look after. | According to ICU guidelines for the UK ^108^. | 1 |
| Beds per G&A Nurse | The maximum threshold of the number of beds that a single G&A nurse could safely look after. | According to Royal College of Nursing guidelines ^109^. | 5 |
| Beds per CC Senior Doctor | The maximum threshold of the number of beds that a single CC senior doctor could safely look after. | According to ICU guidelines for the UK ^108^. | 15 |
| Beds per CC Junior Doctor | The maximum threshold of the number of beds that a single CC junior doctor could safely look after. | According to ICU guidelines for the UK ^108^. | 8 |
| Beds per G&A Senior Doctor | The maximum threshold of the number of beds that a single G&A senior doctor could safely look after. | According to Royal College of Physician guidelines ^110^. | 15 |
| Beds per G&A Junior Doctor | The maximum threshold of the number of beds that a single CC junior doctor could safely look after. | According to Royal College of Physician guidelines ^110^. | 15 |
| COVID-19-related Inputs [cells C20-G20] | | | |
| Number of COVID-19 patients in CC beds | The observed or expected number of COVID-19 patients in CC on any given day.  *If the setting-specific distribution of COVID-19 patients between critical and non-critical (G&A) care is not known, e.g. because no data on COVID-19 hospital admissions are available yet, users can apply reported ratios from other settings as an assumption. For example, in an Italian study, the proportion of COVID-19 hospitalised patients that were admitted to ICU was 16%* ^111^*.* | Maximum observed number of confirmed COVID-19 cases in CC (on 12 April 2020) reported in government daily briefings ^1^. | 3,100 |
| Number of COVID-19 patients in G&A beds | The observed or expected number of COVID-19 patients in G&A care on any given day. | Maximum observed number of confirmed COVID-19 cases in G&A (on 12 April 2020) reported in government daily briefings ^1^. | 15,700 |
| Percentage of COVID-19 CC patients requiring breathing support equipment | The percentage of COVID-19 patients in CC requiring respiratory support.  *Note: If not known, users can use the suggested value of 63%.* | Proxy for the percentage of COVID-19 CC patients requiring a ventilator based on analyses from the Intensive Care National Audit & Research Centre on the needs of UK CC patients within the first 24 hours of admission ^112,113^. | 63% |
| COVID-19-related sickness rate for nurses | The observed or expected percentage of nurses (CC and G&A) on sickness absence due to COVID-19 on any given day.  Note that sickness absence for reasons other than COVID-19 should be accounted for in baseline inputs of existing staff FTEs (see Baseline inputs on existing hospital capacity section). COVID-19 related sickness rates are applied in addition to this in all the staffing calculations on the Output sheet, including the baseline scenario. | Media reports of the estimated overall NHS staff sickness rate, according to the Department of Health and Social Affairs ^114^. | 8.1% |
| COVID-19-related sickness rate for doctors | The observed or expected percentage of doctors (CC and G&A; junior and senior) on sickness absence due to COVID-19 on any given day.  Note that sickness absence for reasons other than COVID-19 should be accounted for in baseline inputs of existing staff FTEs (see Baseline inputs on existing hospital capacity section). COVID-19 related sickness rates are applied in addition to this in all the staffing calculations on the Output sheet, including the baseline scenario. | A recent survey (April 2020) by the Royal College of Physicians asked doctors to estimate recent levels of sickness, broken down into COVID and non-COVID reasons ^114^. | 14% |
| Additional Inputs [cells H20-I20] | | | |
| Headcount to FTE multiplier | Multiplier to convert staff headcounts into staff FTEs. Since official announcements of additional staff numbers are often given in headcounts, the multiplier is provided to convert headcounts into FTEs, as estimated by NHS workforce data ^115^. This can be updated by the user if necessary or omitted if not applicable. | Calculated by dividing the headcount of total NHS staff in December 2019 by total FTE ^115^. | 0.88 |
| Reference population size for output calculation | The spare capacity output is by default calculated in absolute terms. Users can also calculate capacity per 10,000 reference population by inputting a reference population size here. Resetting the reference population to 10,000 will provide the absolute spare capacity (e.g. absolute number of beds spare or deficit). | Estimated population size of England ^116^. | 55,977,200 |

**Appendix Table B2: Description, model assumptions and example values (for England) of the interventions presented in the planner.**

| Intervention | Description | Model Assumptions  *Assumptions to derive input values for England* | England example intervention value |
| --- | --- | --- | --- |
| Cancellation of elective operations | To reduce the number of beds occupied and staff required, non-elective surgeries are cancelled. | - This frees up both CC and G&A beds. - An analysis of the busiest month in hospitals last year (January 2019) using Hospital Episode Statistics estimated the proportion of beds filled with non-emergency, non-maternity and non-cancer related elective patients in CC and G&A beds respectively ^117^. Thus, the proportion of beds freed from cancelling elective surgeries, not of the type above could be estimated. - This was inputted directly as a % change. | - 30% freed beds from CC. - 41% freed beds from G&A. |
| National guidelines for the prioritisation of CC resources | In situations of a severe scarcity of resources, patients are prioritised for CC following official guidelines (triage criteria vary across countries). | - This frees up CC beds. - An analysis of the busiest month in hospitals last year (January 2019) using Hospital Episode Statistics estimated the proportion of beds that could be freed according to the recently published NICE guidelines on CC ^117^. - This was inputted directly as a % change. | - 60% freed beds from CC. |
| Set up of field hospitals | Non-hospital sites are temporarily turned into hospitals during the pandemic. | - The type of beds in field hospitals are known, and if not are assumed to be G&A ^87,118^. | - 500 CC beds. - 8,000 G&A beds. |
| Use of private healthcare resources | National health systems temporarily use private healthcare resources to provide public care. | - Beds are given as a generic figure and distributed according to baseline proportions ^119^. - Numbers of nurses and doctors are given as headcounts and not disaggregated by seniority or bed type. This is distributed according to baseline proportions and multiplied by the FTE multiplier ^119^. - Private hospital operating theatres are converted into CC wards, a number of which are kept open for emergencies ^119,120^. - The number of beds per theatre can be varied but is constant across all theatres. | - 8000 hospital beds. Using proportions of baseline beds this gives 312 CC (4%) and 7,688 G&A (96%). - 1,200 ventilators. - 10,000 nurses. Using proportions of baseline nursing this gives 966 CC (11%) 7,934 and G&A (89%). - 700 doctors. Using proportions of baseline nursing this gives 24 senior CC (4%), 17 junior CC (3%), 321 senior G&A (52%) and 261 junior G&A (42%). - 14 operating theatres in total. Assume 50% of these are converted for use as CC beds. - Assume 2 beds per each theatre. |
| Conversion of operating theatres to CC wards | The space in operating theatres is converted into CC wards with a number of beds. Some operating theatres must be kept operational for emergencies. | - Hospital operating theatres are converted into CC wards, a number of which are kept open for emergencies ^103^. - The number of beds per theatre can be varied but is constant across all theatres. | - 2,404 of operating theatres in England, calculated by assuming teaching and specialist trusts have one Tertiary hospital requiring 5 theatres open for emergency surgery. All other hospitals are assumed to be District general hospital (DGH) and require only 1 theatre open for emergencies. - Assume 2 beds per theatre. |
| Conversion of G&A beds to CC beds | Hospitals increase specialized bed capacity by converting some of their G&A wards into CC wards, which requires investments into specialized staff and equipment. | - A number of beds are taken from G&A and added to CC. The overall number of beds does not change. | - Arbitrary value of 2,000. |
| Upskill G&A staff to work in CC wards | G&A staff and staff from other clinical specialties are given basic training to work in CC wards. | - A number of nurses and junior doctors are taken from G&A and added to CC. The overall number of staff does not change. | - Arbitrary value of 2% for both. This results in the reallocation of 647 nurses and 206 junior doctors. |
| Return of former healthcare staff | Individuals who recently worked in the health system are asked to return for the duration of the pandemic. | - Numbers of nurses and doctors are given as headcounts and not disaggregated by seniority or bed type. This is converted into FTE via the FTE multiplier ^121^. - This is distributed according to baseline proportions. This accounts for the fact that although more retired doctors are likely to be senior, many may not be willing to take on a clinical decision-making position and thus would return as ‘junior’. | - 6,147 nurses which equate to 5471 FTE. Using proportions of baseline nurses this gives 594 CC (11%) and 4,877 G&A (89%). - 2,660 doctors which equate to 2,367 FTE. Using proportions of baseline nursing this gives 93 senior CC (4%), 65 junior CC (3%), 1,220 senior G&A (52%) and 990 junior G&A (42%). |
| Deployment of newly qualified and final year medicine and nursing students | Final year medical and nursing students may have their qualification process accelerated to enable them to start working immediately. | - Numbers of nurses and doctors are given as headcount and thus converted using the FTE multiplier ^121^. - The new nurses and doctors are assumed to be G&A nurses and G&A junior doctors respectively. | - 18,700 nurses which equate to 16,643 FTE G&A nurses. - 5,500 doctors which equate to 4,895 FTE junior G&A doctors. |
| Deployment of international doctors at the final stage of their conversion assessment | Doctors who qualified overseas often must take additional exams to practice in a new country. For those who are close to the end of this process could be accelerated or the final stages waived. | - Numbers of doctors are given as headcount and thus converted using the FTE multiplier ^122^. - These doctors are assumed to be junior G&A doctors. | - 3,000 junior G&A doctors |
| Procurement of newly manufactured respiratory support equipment | Governments purchase additional respiratory support equipment, or request manufacture of devices, to increase the number of machines nationally. | - This is given as a usable figure and thus no further calculations are required ^65^. | - 20,000 ventilators. |

**Appendix Table B3: Overview of the outputs as presented in *Output (Numeric)* in the planner.**

| **Output variable** | **Description** | **Interpretation** |
| --- | --- | --- |
| **Spare capacity [columns C-K]** | For each intervention, the spare capacity of beds, respiratory support equipment, and staff is calculated by subtracting the capacity required (as described in Appendix A Section 5 and Appendix Table A1) to care for the given number of COVID and non-COVID patients from the total capacity. For example, the latter involves subtracting the number of occupied beds from the total number of beds, and the number of staff FTEs needed to service these occupied beds from the total staff FTEs. If the users requires spare capacity in terms of per 10,000 population, the spare capacity is then divided by the input reference population chosen by the user and presented as per 10,000 population. | Negative values for spare capacity represent a **deficit** of this resource and are highlighted in red. The relationship between inputs and outputs is further illustrated in Appendix Figure A1, highlighting that a deficit occurs if the capacity required to care for all COVID and non-COVID patients exceeds the total capacity. |
| **Per cent change in spare capacity compared to baseline [columns L-T]** | The per cent change in spare capacity compared to baseline allows comparing the effect of different interventions on the spare capacity of beds, respiratory support equipment, and staff for a given number of patients. This is defined as in Equation (1). | Positive percentages represent an improvement in spare capacity in the respective intervention compared to baseline; negative percentages highlight a reduction in spare capacity compared to baseline, and a 0% change means the intervention has not affected a given spare capacity output compared to baseline. Percentages can be compared across interventions to assess which intervention leads to the most substantial change. |
| **Staff planner: staff required for total bed numbers [columns U-Z]** | The minimum number of each type of staff required per total bed is based on the maximum ratio input as part of the baseline. This is compared to the current total number of staff in columns AH, AJ, AL, AN, AP and AR, which has been adjusted down based on the inputted COVID-related sickness rates. | If this is green, the current total number of staff is sufficient to treat all patients if beds reached capacity. Red means the opposite and indicates that further staff would be required to fully implement this intervention under the specified ratios. |
| **Staff ratio check for current patients [columns AJ, AL, AN, AP, AR, AT]** | The tool also provides a check as to whether the staff numbers required per occupied bed are satisfactory, again based on the maximum ratio input as part of the baseline. This is compared to the current total number of staff, which has been adjusted down based on the inputted COVID-related sickness rates. | If this is green, it means that the current total number of staff is sufficient to treat all patients currently in beds only. Red means the opposite and indicates that further staff would be required to treat current patients safely that intervention under the specified ratios. |

## References

1. Stevens S, Pritchard A. IMPORTANT AND URGENT – NEXT STEPS ON NHS RESPONSE TO COVID-19. Published online March 17, 2020. https://www.england.nhs.uk/coronavirus/wp-content/uploads/sites/52/2020/03/20200317-NHS-COVID-letter-FINAL.pdf

2. Nowotny M. Operationen verschieben, statt Risiken eingehen. *Ö1 Wissenschaft*. https://science.orf.at/stories/3200396/. Published March 24, 2020.

3. Coronavirus: l’Etat mobilise tous les hôpitaux généraux du pays. *La Libre*. https://www.lalibre.be/planete/sante/coronavirus-l-etat-mobilise-tous-les-hopitaux-generaux-du-pays-5e69d9cef20d5a29c65396c6. Published March 12, 2020.

4. Sundhedsstyrelsen. Håndtering af COVID-19: Prognose og kapacitet i Danmark for in-tensiv terapi. Published online 2020. https://www.sst.dk/-/media/Nyheder/2020/ITA_COVID_19_220320.ashx?la=da&hash=633349284353F4D8559B231CDA64169D327F1227

5. Teuch J-J, Gangloff A, Di Fiore F, et al. Stratégie pour la pratique de la chirurgie digestive et oncologique en situation d’épidémie de COVID 19. *J Chir Viscerale*. Published online March 19, 2020. https://www.elsevier.com/fr-fr/connect/chirurgie/strategie-pour-la-pratique-de-la-chirurgie-digestive-et-oncologique-en-situation-depidemie-de-covid-19

6. Die Bundesregierung. Besprechung der Bundeskanzlerin mit den Regierungschefinnen und Regierungschefs der Länder am 12. März 2020. Published March 12, 2020. https://www.bundesregierung.de/breg-de/themen/coronavirus/beschluss-zu-corona-1730292

7. Minstero della Salute. COVID-19, i consigli dell’ISS in attesa dell’intervento di artroprotesi dell’anca o del ginocchio. Published March 25, 2020. http://www.salute.gov.it/portale/nuovocoronavirus/dettaglioNotizieNuovoCoronavirus.jsp?lingua=italiano&menu=notizie&p=dalministero&id=4320

8. Kinsch F, Camposeo M. Wéi hunn d’Spideeler sech preparéiert an organiséiert? *RTL Luxembourg*. https://www.rtl.lu/news/national/a/1490885.html. Published March 27, 2020.

9. Den Norske Legeforening. Legeforeningens fagmedisinske foreninger har laget prioriteringsråd i forbindelse med koronavirus-epidemien. Published 2020. https://www.legeforeningen.no/fag/aktuelt/fagmedisinske-foreningenes-prioriteringer-i-forbindelse-med-koronaviruset/#100498

10. Sevillano EG. Hospitales privados ya han empezado a cancelar operaciones que no son urgentes por el coronavirus. *El Pais*. https://elpais.com/sociedad/2020-03-10/hospitales-privados-ya-han-empezado-a-cancelar-operaciones-que-no-son-urgentes-por-el-coronavirus.html. Published March 10, 2020.

11. Nationella principer för prioritering inom intensivvård under extraordinära förhållanden. Published online 2020. https://www.socialstyrelsen.se/globalassets/sharepoint-dokument/dokument-webb/ovrigt/nationella-prioriteringar-intensivarden.pdf

12. Aschwanden E. Chaos um verschiebbare Eingriffe: Die Spitäler operieren weiter, der oberste Chirurg warnt seine Kollegen vor den Folgen. *Neue Zurcher Zeitung*. https://www.nzz.ch/schweiz/verwirrung-um-wahleingriffe-wer-darf-in-der-corona-krise-noch-operiert-werden-ld.1547395?reduced=true%20. Published March 20, 2020.

13. NICE. NICE updates rapid COVID-19 guidelines on critical care. Published April 3, 2020. Accessed April 3, 2020. https://www.nice.org.uk/news/article/nice-updates-rapid-covid-19-guideline-on-critical-care

14. Österreichischen Gesellschaft für Anästhesiologie, Reanimation und Intensivmedizin. Allokation intensivmedizinischer Ressourcen aus Anlass der Covid-19-Pandemie. Published online 2020. https://www.oegari.at/web_files/cms_daten/covid-19_ressourcenallokation_gari-statement_v1.7_final_2020-03-17.pdf

15. Salaun T, Irish J. Army steps in to help hospitals in east France fight coronavirus. *Reuters*. https://uk.reuters.com/article/uk-health-coronavirus-france-army/army-steps-in-to-help-hospitals-in-east-france-fight-coronavirus-idUKKBN2151OT. Published March 18, 2020.

16. Muller N. Coronavirus: German doctors lay down life-or-death guidelines. *DW*. https://www.dw.com/en/coronavirus-german-doctors-lay-down-life-or-death-guidelines/a-52925064. Published March 26, 2020.

17. Vergano M, Bertolini G, Giannini A, et al. Raccomandazioni di etica clinica per l’ammissione a trattamenti intensivi e per la loro sospensione, in condizioni eccezionali di squilibrio tra necessità e risorse disponibili. Published online 2020. http://www.siaarti.it/SiteAssets/News/COVID19%20-%20documenti%20SIAARTI/SIAARTI%20-%20Covid19%20-%20Raccomandazioni%20di%20etica%20clinica.pdf

18. Sevillano EG, Guell O. Spanish doctors prepare to make difficult decisions if intensive care units reach capacity. *El Pais*. https://english.elpais.com/society/2020-03-20/spanish-doctors-prepare-to-make-difficult-decisions-if-intensive-care-units-reach-capacity.html. Published March 20, 2020.

19. Bateman J. ‘Solidarity knows no borders’: Germany treating dozens of coronavirus patients from Italy and France. *The Independent*. https://www.independent.co.uk/news/world/europe/coronavirus-germany-italy-france-hospital-treatment-covid-19-a9440906.html. Published April 2, 2020.

20. Scherndl G. Wie man das Coronavirus vom Altersheim fernhalten will. *Der Standard*. https://www.derstandard.at/story/2000115675544/wie-man-das-coronavirus-vom-altersheim-fernhalten-will. Published March 13, 2020.

21. Pensionsversicherungsanstalt. Maßnahmen zur Eindämmung des Coronavirus im Bereich der PVA. Published April 8, 2020. https://www.pensionsversicherung.at/cdscontent/?contentid=10007.857666&portal=pvaportal%20

22. COVID-19 Health Policy Response Monitor: Policy responses for Germany. Published March 31, 2020. https://www.covid19healthsystem.org/countries/germany/livinghit.aspx?Section=2.2%20Workforce&Type=Section

23. Zeimetz C, Hochard C. Rehazenter vu Croix-Rouge prett, 1. Corona-Patient ukomm. *RTL Luxembourg*. https://www.rtl.lu/news/national/a/1490735.html. Published March 27, 2020.

24. Ministry of Health, Government of Luxembourg. Opening of the 4 new Advanced care centres. Published March 26, 2020. https://msan.gouvernement.lu/en/actualites.gouvernement%2Ben%2Bactualites%2Btoutes_actualites%2Bcommuniques%2B2020%2B03-mars%2B26-ouverture-csa.html

25. Karkov R. Få intensivpladser, men godt coronaberedskab i Danmark. *Berlingske*. https://www.berlingske.dk/samfund/faa-intensivpladser-men-godt-coronaberedskab-i-danmark. Published March 8, 2020.

26. Mirguet O. Coronavirus : l’hôpital de campagne à Mulhouse sera prêt ce lundi. *La Tribune*. https://www.latribune.fr/economie/france/coronavirus-l-hopital-de-campagne-prend-forme-a-mulhouse-842971.html. Published 2020.

27. Baldi C. Coronavirus, inaugurato l’ospedale Fiera a Milano: la più grande terapia intensiva d’Italia.https://www.lastampa.it/milano/2020/03/31/news/coronavirus-inaugurato-l-ospedale-fiera-a-milano-la-piu-grande-terapia-intensiva-d-italia-1.38660553?refresh_ce%20https://www.agensir.it/italia/2020/03/16/coronavirus-a-roma-inaugurato-il-covid-2-hospital-al-columbus-trasferiti-i-primi-pazienti/. Published March 31, 2020.

28. Centre Hospitalier de Luxembourg. Les premiers éléments du « Field Hospital » ont été mis en service ce matin à 7h00. Published 03 2020. https://www.chl.lu/fr/actualites/les-premiers-elements-du-field-hospital-mis-en-service-30-mars-2020

29. Kalveland J. FHI om sykehusbygg: – Gammeldags har blitt nymotens. *Dagens Medisin*. https://www.dagensmedisin.no/artikler/2020/03/30/fhi-stotter-uttalelse-om-sykehus-med-hoye-tarn--gammeldags-har-blitt-nymotens/. Published March 30, 2020.

30. Kassam A. “We are naked against the virus”: tales of despair from Spain’s hospital frontline. *The Guardian*. https://www.theguardian.com/world/2020/mar/29/coronavirus-madrid-doctor-hospital-desperate-supplies. Published March 29, 2020.

31. Stockholm hospitals brace for “increasing coronavirus storm.” *The Local*. https://www.thelocal.se/20200330/pressure-increases-on-stockholm-hospitals-as-coronavirus-storm-increases-in-strength. Published March 30, 2020.

32. Schweizer Armee. Armee und Zivilschutz reaktivieren das alte Spital-Bettenhaus. Published March 25, 2020. https://www.vtg.admin.ch/de/aktuell/coronavirus.detail.news.html/vtg-internet/verwaltung/2020/20-03/armee-und-zivilschutz-reaktivieren-das-alte-spital-bettenhaus.html

33. NHS England. New NHS nightingale hospital to fight coronavirus. Published March 24, 2020. https://www.england.nhs.uk/2020/03/new-nhs-nightingale-hospital-to-fight-coronavirus/

34. COVID-19 i Danmark – 30. marts 2020. Published online 2020. https://www.sst.dk/-/media/Udgivelser/2020/Corona/Status-og-strategi/Status_COVID19_femte-uge.ashx?la=da&hash=2889D7E5580B1450EB896A3A1EB69A1E4ADA93F6

35. Coronavirus/ Aiop: da ospedali privati 1.300 posti in terapia intensiva. *Sanita24*. https://www.sanita24.ilsole24ore.com/art/imprese-e-mercato/2020-03-20/coronavirus-aiop-ospedali-privati-1300-posti-terapia-intensiva-132254.php?uuid=ADqJTjE. Published March 20, 2020.

36. Den Norske Legeforening. Om smittefare, tilrettelegging og omplassering i forbindelse med korona-utbruddet. Published 2020. https://www.legeforeningen.no/politikk-og-samfunn/informasjon-om-koronaviruset/ofte-stilte-sporsmal-om-smittefare/#98947

37. Henley J, Willsher K, Kassam A. Coronavirus: France imposes lockdown as EU calls for 30-day travel ban. *The Guardian*. https://www.theguardian.com/world/2020/mar/16/coronavirus-spain-takes-over-private-healthcare-amid-more-european-lockdowns. Published March 16, 2020.

38. Aschwanden E, Jankovsky P. Das Tessin verlegt erste Corona-Patienten in Deutschschweizer Spitäler. *Neue Zurcher Zeitung*. https://www.nzz.ch/schweiz/tessin-verlegt-erste-corona-patienten-in-deutschschweizer-spitaeler-ld.1549417?reduced=true. Published March 31, 2020.

39. Personal Communication: email correspondence with Danish Government. Published online April 3, 2020.

40. Spinney L. “We are ready”: inside the Paris hospitals bracing for coronavirus. *The Guardian*. https://www.theguardian.com/world/2020/mar/16/we-are-ready-inside-the-paris-hospitals-bracing-for-coronavirus-covid-19-france. Published March 16, 2020.

41. Kiss P, Schmid-Johannsen J, Schafer J, Heiliger N, Lang U. So viele COVID-19-Patienten können wir beatmen.https://www.swr.de/swraktuell/intensivstationen-am-limit-100.html%20. Published 04 2020.

42. Tondo L. Italian hospitals short of beds as coronavirus death toll jumps. *The Guardian*. https://www.theguardian.com/world/2020/mar/09/italian-hospitals-short-beds-coronavirus-death-toll-jumps. Published March 9, 2020.

43. Benavides L. Spain’s Health Staff Are Catching The Coronavirus As Protective Gear Runs Short. Published March 31, 2020. https://www.npr.org/sections/coronavirus-live-updates/2020/03/31/824654965/spains-health-staff-are-catching-the-coronavirus-as-protective-gear-runs-short?t=1585736417975&t=1586325599901

44. Honegger L. Corona-Virus: Schweizer Intensivstationen wappnen sich für das schlimmste Szenario. *Aargauer Zeitung*. https://www.aargauerzeitung.ch/schweiz/corona-virus-schweizer-intensivstationen-wappnen-sich-fuer-das-schlimmste-szenario-136989579. Published March 12, 2020.

45. Personal Communication: discussion with doctors at Imperial College Healthcare Trust. Published online March 27, 2020.

46. Ministry of Health and Social Affairs, Government Offices of Sweden. Work in the areas of public health, medical care, social care and social insurance with regard to the COVID-19 virus. Published March 20, 2020. https://www.government.se/articles/2020/03/s-work-in-the-areas-of-public-health-medical-care-social-care-and-social-insurance-with-regard-to-the-covid-19-virus/

47. Coronavirus: le réseau hospitalier bruxellois loin d’être saturé grâce au confinement précoce. *rtbr*. https://www.rtbf.be/info/societe/detail_coronavirus-le-reseau-hospitalier-bruxellois-loin-d-etre-sature-grace-au-confinement-precoce?id=10463661. Published February 20, 2020.

48. Irish J, Pennetier M. France extends lockdown to April 15 as coronavirus wave swamps Paris. *Reuters*. https://www.reuters.com/article/us-health-coronavirus-france/france-extends-lockdown-to-april-15-as-coronavirus-wave-swamps-paris-idUSKBN21E1AT. Published March 27, 2020.

49. Governo Italiano Presidenza del Consiglio dei Ministri. Decreto-legge 17 marzo 2020. Published March 18, 2020. http://www.governo.it/it/articolo/decreto-legge-17-marzo-2020/14333

50. Forster C. Ohne Gegenmassnahmen kommen auch die Schweizer Spitäler bei der Behandlung von Corona-Erkrankten schnell an ihre Grenzen. *Neue Zurcher Zeitung*. https://www.nzz.ch/schweiz/coronavirus-schweizer-spitaelern-droht-der-kollaps-ld.1546008?reduced=true. Published March 13, 2020.

51. Italia Emergenzia. 7 - CORSO DI VENTILAZIONE MECCANICA ”LE CURVE CHE TOLGONO IL FIATO”. Published 2016. http://www.italiaemergenza.com/corsi-formazione.do?7%20%2D%20CORSO%20DI%20VENTILAZIONE%20MECCANICA%20%22LE%20CURVE%20CHE%20TOLGONO%20IL%20FIATO%22&key=1458741056&dettagli=y&sort_on=8&search_for=!X1

52. Berg TL. Nye opplysninger: Norge har færre enn 1400 sykehusplasser for korona-syke. *Filter Nyheter*. https://filternyheter.no/korona-norge-forbereder-epidemi-har-maks-1400-sengeplasser-for-alvorlig-syke/. Published March 7, 2020.

53. Pensionierte Ärzte sollen Salzburger Patienten in häuslicher Quarantäne versorgen Author: Salzburger Nachrichten. *Salzburger Nachrichten*. https://www.sn.at/salzburg/chronik/pensionierte-aerzte-sollen-salzburger-patienten-in-haeuslicher-quarantaene-versorgen-84693256. Published March 11, 2020.

54. Ministere des Solidarites et de la Sante. COVID-19 : Appel à volontariat auprès des professionnels de santé. Published March 25, 2020. https://solidarites-sante.gouv.fr/actualites/actualites-du-ministere/article/covid-19-appel-a-volontariat-aupres-des-professionnels-de-sante

55. Governo Italiano. Misure di potenziamento del Servizio sanitario nazionale e di sostegno economico per famiglie, lavoratori e imprese connesse all’emergenza epidemiologica da COVID-19. Published 2020. http://www.governo.it/it/articolo/decreto-legge-17-marzo-2020/14333

56. Le Gouvernement du Grand-Duche de Luxembourg. Professionnels de santé: inscription obligatoire jusque mardi à minuit. Published March 23, 2020. https://sante.public.lu/fr/actualites/2020/03/inscription-professionnels-sante/index.html

57. Helsedirektoratet. Registrering og formidling av ledig helsepersonell. Published March 24, 2020. https://www.helsedirektoratet.no/tema/beredskap-og-krisehandtering/koronavirus/okt-behov-for-helsepersonell-under-koronavirusutbruddet

58. Ministerio de Sanidad, Consumo y Bienestar Social. El Ministerio de Sanidad establece medidas para el refuerzo de personal sanitario y el suministro de material. Published March 15, 2020. https://www.mscbs.gob.es/gabinete/notasPrensa.do?id=4812

59. Hagman J. Så rustar Blekinge för brist på vårdpersonal. *Svt Nyheter*. https://www.svt.se/nyheter/lokalt/blekinge/sa-rustar-blekinge-for-brist-pa-vardpersonal. Published April 1, 2020.

60. Altermatt S. Auch Studierende und Pensionierte helfen mit: So rüsten sich Schweizer Spitäler in der Corona-Krise gegen den Kollaps. *Tagblatt*. https://www.tagblatt.ch/schweiz/auch-studierende-und-pensionierte-helfen-mit-so-ruesten-sich-schweizer-spitaeler-in-der-corona-krise-gegen-den-kollaps-ld.1204386. Published March 16, 2020.

61. NHS England. Former docs and nurses told “Your NHS Needs You” to tackle greatest global health threat in history. Published March 19, 2020. https://www.england.nhs.uk/2020/03/former-docs-and-nurses-told-your-nhs-needs-you-to-tackle-greatest-global-health-threat-in-history/

62. General Medical Council. Information for medical students. Published 2020. https://www.gmc-uk.org/news/news-archive/coronavirus-information-and-advice/information-for-medical-students

63. Royal College of Nursing. COVID-19: actions announced to expand the nursing workforce. Published March 19, 2020. https://www.rcn.org.uk/news-and-events/news/uk-covid-19-actions-announced-to-expand-the-nursing-workforce-190320

64. Ministerio de Sanidad, Consumo y Bienestar Social. El Gobierno impulsa la contratación de cerca de 200 profesionales extranjeros en situación regular del sector sanitario ante la crisis del Covid-19. Published March 27, 2020. https://www.mscbs.gob.es/gabinete/notasPrensa.do?id=4828

65. Coronavirus: How easy it for the UK to make more ventilators? *BBC News*. https://www.bbc.co.uk/news/technology-51909812. Published March 17, 2020.

66. Medinlive Medizinische Information Live. Autobauer sollen Beatmungsgeräte bauen. Published March 20, 2020. https://www.medinlive.at/wissenschaft/autobauer-sollen-beatmungsgeraete-bauen

67. Fagniez P. Coronavirus: un respirateur pour plusieurs patients, ça fonctionne vraiment? *RTL Info*. https://www.rtl.be/info/vous/temoignages/coronavirus-un-respirateur-pour-plusieurs-patients-ca-fonctionne-vraiment--1207846.aspx. Published March 28, 2020.

68. Fabrication de matériel critique en 3D pour les soignants et les patients rendue possible par le soutien de Kering. *Assistance publique – Hôpitaux de Paris*. https://www.aphp.fr/contenu/fabrication-de-materiel-critique-en-3d-pour-les-soignants-et-les-patients-rendue-possible. Published 2020.

69. Bucke T, Ghiglione D. European countries search for ventilators as virus cases surge. *The Financial Times*. https://www.ft.com/content/5a2ffc78-6550-11ea-b3f3-fe4680ea68b5. Published 2020.

70. Pollina E, Piovaccari G. Exclusive: Ferrari and Fiat look at helping Italy make ventilators in coronavirus crisis. *Reuters*. https://www.reuters.com/article/us-health-coronavirus-ventilators-italy/exclusive-ferrari-and-fiat-look-at-helping-italy-make-ventilators-in-coronavirus-crisis-idUSKBN2162YT. Published March 19, 2020.

71. Tasch O. Le Luxembourg bien équipé face à la pandémie. *Luxembourger Wort*. https://www.wort.lu/fr/luxembourg/le-luxembourg-bien-equipe-face-a-la-pandemie-5e6f847ada2cc1784e358d5f. Published March 16, 2020.

72. Martinsen E, Ali I, Bratten MT. Sykehus frykter nye korona-respiratorer kommer for sent. *NRK*. https://www.nrk.no/osloogviken/korona_-slik-jobber-sykehusene-for-a-lose-respirator-krisen-1.14961860. Published March 27, 2020.

73. Banco Bilbao Vizcaya Argentaria. BBVA-sourced medical supplies to fight coronavirus arrive in Spain. Published April 1, 2020. https://www.bbva.com/en/bbva-sourced-medical-supplies-to-fight-coronavirus-arrive-in-spain/

74. Socialdepartementet, Regeringskansliet. Pressträff med socialminister Lena Hallengren. Published March 19, 2020. https://www.regeringen.se/pressmeddelanden/2020/03/presstraff-med-socialminister-lena-hallengren1/

75. Strobl R, Neumann M, Greil A, et al. Coronavirus in Österreich: Kanzler Kurz spricht von einschneidender Maßnahme - Neuer „Fahrplan“ soll Covid-19 eindämmen. *Merkur.de*. https://www.merkur.de/welt/corona-oesterreich-ischgl-kurz-aktuell-statistik-karte-faelle-zahlen-hoffnung-tote-infizierte-zr-13633041.html. Published April 7, 2020.

76. Istituto Superiore di Sanità (ISS). Procedure per richiesta produzione mascherine. Published 2020. http://www.iss.it/procedure-per-richiesta-produzione-mascherine

77. Regjeringen. Lettere å få smittevernutstyr til Norge. Published 2020. https://www.regjeringen.no/no/aktuelt/lettere-a-fa-smittevernutstyr-til-norge/id2694834/

78. Gap, Zara start producing masks and other supplies for first responders. *Today*. Published online March 23, 2020. https://www.today.com/style/hanes-zara-start-producing-masks-other-supplies-first-responders-t176595

79. Socialstyrelsen. Ny förordning ger Socialstyrelsen mandat att besluta om regioners och kommuners resursanvändning. Published March 19, 2020. https://www.socialstyrelsen.se/aktuellt/ny-forordning-ger-socialstyrelsen-mandat-att-besluta-om-regioners-och-kommuners-resursanvandning/

80. Willet K. Guidance on supply and use of PPE. Published online March 20, 2020. https://www.england.nhs.uk/coronavirus/wp-content/uploads/sites/52/2020/03/PPE-Letter-FINAL-20-March-2020-updated-on-22-March-2020.pdf

81. Federal Public Service Health, Food Chain and Environment. Delivery and distribution of masks: situation and stocks. Published March 25, 2020. https://www.info-coronavirus.be/en/news/delivery-and-distribution-of-masks/

82. Lagemiddel Styrelsen. Lægemiddelstyrelsen får flere beføjelser til at modvirke forsyningsproblemer. Published March 24, 2020. https://laegemiddelstyrelsen.dk/da/nyheder/2020/laegemiddelstyrelsen-faar-flere-befoejelser-til-at-modvirke-forsyningsproblemer/

83. Thompson R. Coronavirus: French protective mask manufacturer scraps NHS order to keep masks in France. *Euronews*. https://www.euronews.com/2020/03/06/coronavirus-french-protective-mask-manufacturer-scraps-nhs-order-to-keep-masks-in-france. Published March 6, 2020.

84. European Commission. Draft Decree relating to the necessary requisitions in the context of the fight against the COVID-19 virus and repealing Decree No 2020-190 of 3 March 2020. Published 2020. https://ec.europa.eu/growth/tools-databases/tris/en/search/?trisaction=search.detail&year=2020&num=126

85. Utbruddsgruppa ved Folkehelseinstituttet. COVID-19-EPIDEMIEN: Risikovurdering og respons i Norge (Versjon 3). Published online 2020. https://www.fhi.no/contentassets/c9e459cd7cc24991810a0d28d7803bd0/notat-om-risiko-og-respons-2020-03-12.pdf

86. Bernal Delgado E, Angulo Pueyo E, Estupinan Romero F. COVID-19 Health Policy Response Monitor: Policy responses for Spain. COVID-19 Health System Response Monitor. Published April 1, 2020. https://www.covid19healthsystem.org/countries/spain/countrypage.aspx

87. Rajan S. Covid-19 Health System Response Monitor - United Kingdom. Published online March 30, 2020. https://www.covid19healthsystem.org/countries/unitedkingdom/countrypage.aspx

88. Bundesministerium fur Gesundheit. Gemeinsamer Krisenstab BMI/BMG fällt weitere Beschlüsse. Published March 4, 2020. https://www.bundesgesundheitsministerium.de/weiterere-beschluesse-krisenstab-bmi-bmg.html

89. Guarascio F, Blenkinsop P. EU fails to persuade France, Germany to lift coronavirus health gear controls. *Reuters*. https://uk.reuters.com/article/us-health-coronavirus-eu/eu-fails-to-persuade-france-germany-to-lift-coronavirus-health-gear-controls-idUKKBN20T166. Published March 6, 2020.

90. Department of Health and Social Care. Guidance: Personal protective equipment (PPE): export control process. Published March 15, 2020. https://www.gov.uk/government/publications/personal-protective-equipment-ppe-export-control-process/personal-protective-equipment-ppe-export-control-process

91. Minstero degli Affari Esteri e della Cooperazione Internazionale. Export Authorisation - Personal Protective Equipment. Published March 19, 2020. https://www.esteri.it/mae/en/sala_stampa/archivionotizie/approfondimenti/2020/03/autorizzazione-all-esportazione-dispositivi-di-protezione-individuale.html

92. Verordnung über Massnahmen im Bereich der Stellenmeldepflicht im Zusammenhang mit dem Coronavirus. Published online 2020. https://www.admin.ch/opc/de/official-compilation/2020/1071.pdf

93. Federale Overheidsdienst Volksgezondheid, Veiligheid van d Voedselketen en Leefmilieu. Covid-19 – Federale financiële steun voor de ziekenhuizen. Published March 20, 2020. https://www.health.belgium.be/nl/news/covid-19-federale-financiele-steun-voor-de-ziekenhuizen

94. Bundesministerium für Gesundheit. Bundesrat stimmt Gesetzespaketen zur Unterstützung des Gesundheitswesens bei der Bewältigung der Corona-Epidemie zu. Published 2020. https://www.bundesgesundheitsministerium.de/presse/pressemitteilungen/2020/1-quartal/corona-gesetzespaket-im-bundesrat.html

95. Minstero della Salute. Covid-19, in Gazzetta ufficiale il decreto per il potenziamento del Ssn. Published March 10, 2020. http://www.salute.gov.it/portale/nuovocoronavirus/dettaglioNotizieNuovoCoronavirus.jsp?lingua=italiano&id=4188

96. Department for International Development et al. PM announces new funding in fight against spread of coronavirus. Published March 6, 2020. https://www.gov.uk/government/news/pm-announces-new-funding-in-fight-against-spread-of-coronavirus

97. Amazonas Explorer. The latest on COVID-19 coronavirus in Peru. *Amazonas Explorer*. https://amazonas-explorer.com/is-there-coronavirus-in-peru/#13th_April_Medical_staff_over_60_years_old_or_with_medical_problems_to_be_retired. Published June 9, 2020.

98. Dey S. Covid-19: Govt mulls roping in 5th-year medical students, retired doctors to fight coronavirus. *Times of India*. https://timesofindia.indiatimes.com/india/govt-mulls-roping-in-5th-year-med-students-retired-doctors-to-fight-virus/articleshow/74838162.cms. Published March 27, 2020.

99. Oni T. This is the best time to plan for urban Africa’s next health emergency. *Quartz Africa*. https://qz.com/africa/1839019/covid-19-how-to-plan-for-africas-next-health-emergency/. Published April 16, 2020.

100. Xinhua. Turkey to build 2 hospitals in Istanbul for COVID-19 patients. *Xinhua*. http://www.xinhuanet.com/english/2020-04/07/c_138952214.htm. Published April 7, 2020.

101. Roper K. How will SA deal with the peak Covid-19 infections? Here are some options. *City Press*. https://www.news24.com/citypress/Voices/how-will-sa-deal-with-the-peak-covid-19-infections-here-are-some-options-20200415. Published April 15, 2020.

102. NHS Digital. Hospital Episode Statistics (HES). Published 2019. https://digital.nhs.uk/data-and-information/data-tools-and-services/data-services/hospital-episode-statistics#

103. NHS Digital. Critical Care Bed Capacity and Urgent Operations Cancelled. Published 2020. https://www.england.nhs.uk/statistics/statistical-work-areas/critical-care-capacity/

104. NHS Digital. Bed Availability and Occupancy. Published 2020. https://www.england.nhs.uk/statistics/statistical-work-areas/bed-availability-and-occupancy/

105. Shahin J, Harrison DA, Rowan KM. Is the volume of mechanically ventilated admissions to UK critical care units associated with improved outcomes? *Intensive Care Med*. 2014;40(3):353-360. doi:10.1007/s00134-013-3205-4

106. Neville S. Bed-cutting policy leaves NHS short of resources. *The Financial Times*. https://www.ft.com/content/5da1906e-6a1f-11ea-800d-da70cff6e4d3. Published March 21, 2020.

107. NHS. Electronic Staff Records Data. Published online 2019.

108. The Faculty of Intensive Care Medicine. Core Standards for Intensive Care Units. Published online 2013. https://www.ficm.ac.uk/sites/default/files/Core%20Standards%20for%20ICUs%20Ed.1%20(2013).pdf

109. Royal College of Nursing. Setting appropriate ward nurse staffing levels. Published online September 18, 2006. https://www.rcn.org.uk/about-us/policy-briefings/pol-1506#tab1

110. Royal College of Physicians. Guidance on safe medical staffing. Published online July 13, 2018. https://www.rcplondon.ac.uk/projects/outputs/safe-medical-staffing

111. Grasselli G, Pesenti A, Cecconi M. Critical Care Utilization for the COVID-19 Outbreak in Lombardy, Italy: Early Experience and Forecast During an Emergency Response. *JAMA*. Published online March 13, 2020. doi:10.1001/jama.2020.4031

112. Intensive Care National Audit & Research Centre (ICNARC). ICNARC report on COVID-19 in critical care. Published online April 4, 2020. https://www.icnarc.org/About/Latest-News/2020/04/04/Report-On-2249-Patients-Critically-Ill-With-Covid-19,

113. Intensive Care National Audit & Research Centre (ICNARC). ICNARC report on COVID-19 in critical care. Published online May 29, 2020. https://www.icnarc.org/DataServices/Attachments/Download/8419d345-c7a1-ea11-9126-00505601089b

114. Booth R. Number of NHS doctors off sick “may be nearly triple the official estimate.” *The Guardian*. https://www.theguardian.com/world/2020/apr/05/number-nhs-doctors-off-sick-may-be-three-times-more-than-thought. Published April 5, 2020.

115. NHS Digital. NHS Workforce Statistics December 2019. Published online March 26, 2020. https://digital.nhs.uk/data-and-information/publications/statistical/nhs-workforce-statistics/december-2019

116. Office for National Statistics. Population estimates. Published June 29, 2019. https://www.ons.gov.uk/peoplepopulationandcommunity/populationandmigration/populationestimates#timeseries

117. Redaniel T, Savovic J. Trends from the London trusts about whether their medical / surgical and other specialty admissions have increased as the outbreak has developed. Published online April 6, 2020. https://arc-w.nihr.ac.uk/Wordpress/wp-content/uploads/2020/05/BNSSG-COV.15-Trends-from-the-London-Trusts-medical-surgical-and-other-admissions.pdf

118. NHS England. NHS to build more Nightingale hospitals, as London set for opening. Published April 3, 2020. https://www.england.nhs.uk/2020/04/nhs-to-build-more-nightingale-hospitals-as-london-set-for-opening/

119. NHS England. NHS strikes major deal to expand hospital capacity to battle coronavirus. Published March 21, 2020. https://www.england.nhs.uk/2020/03/nhs-strikes-major-deal-to-expand-hospital-capacity-to-battle-coronavirus/

120. NHS England. Supporting Facilites Data.https://www.england.nhs.uk/statistics/statistical-work-areas/cancelled-elective-operations/supporting-facilities-data/. Published 2019.

121. Skelton J, Cannon, Matt, Jurejko J. BBC News - Live Reporting. BBC News. Published March 24, 2020. https://www.bbc.co.uk/news/live/world-52013888/page/2

122. General Medical Council. Recent pass rates for PLAB1 and PLAB2. Published 2020. https://www.gmc-uk.org/registration-and-licensing/join-the-register/plab/recent-pass-rates-for-plab-1-and-plab-2

1. Flag icons downloaded free of charge from <https://www.countryflags.com/en/>, part of ProFlags BV [↑](#footnote-ref-1)
